# Effect of ultrasonic treatment on the quality of Mianning ham

**DOI:** 10.3389/fnut.2023.1199279

**Published:** 2023-08-08

**Authors:** Jiaju He, Wei Wang, Jiamin Zhang, Yanli Zhu, Wenli Wang, Ting Bai, Lili Ji, Lin Chen

**Affiliations:** Key Laboratory of Meat Processing of Sichuan Province, Chengdu University, Chengdu, China

**Keywords:** ultrasonic, desalting technology, Mianning ham, response surface methodology, oral processing

## Abstract

This paper investigates the optimal process for ultrasonic desalination of Mianning ham. The study analyzed various factors such as ultrasonic treatment time, temperature, and power to determine their impact on the rate of desalination of hams. A single factor test was conducted to study the rate of desalination. Further, A Box-Behnken experimental design was used to evaluate the effect of Mianning ham desalination. The design examined the impacts of ultrasound on the physicochemical properties, texture, and sensory of the ham. Response surface processing group underwent oral processing to determine the optimal ultrasonic treatment conditions with the highest acceptance level. The results show that the best conditions were: ultrasonic time 84.56 min, ultrasonic temperature 40.35°C, and ultrasonic power 150.85 W. The average desalination rate of the ham under the optimal conditions was 25.93% ± 0.69%, and the hardness was 4.48 N ± 0.62 N. Overall, this process significantly improved the desalination rate, texture, and sensory quality of Mianning ham, providing solid theoretical support for desalination processing at the back end of ham.

## 1. Introduction

Dry-cured ham is a meat product made by curing, washing, drying, and fermenting the hind legs of pork (1). One of the top ten renowned hams in China is Mianning ham, produced in Mianning County, Liangshan Prefecture, Sichuan Province, China. It is unique because it is made of hind legs from high-quality Liangshan Wujin pigs and does not contain nitrite during the fermentation process. Mianning ham is known for its tender texture, thin skin, bright red color, and unique cured flavor, which makes it easy to preserve (2).

Excessive dietary intake of salt is a global health issue, and according to the World Health Organization (WHO), adults’ daily salt intake should be less than 5 g per day. However, most people consume between 5 and 12 g of salt each day. If salt consumption can be limited to the recommended levels, the WHO approximates that globally, 2.5 million deaths could be avoided yearly (3). Unfortunately, a lot of salt must be used in ham processing to boost protein properties, enhance texture and flavor, and prevent ham spoilage by microorganisms (4, 5). Lowering the salt content in cured ham enhances protein hydrolysis and modifies ham texture, hue, and flavor (6).

Ultrasonic waves can effectively reduce the curing time and accelerate salt diffusion in meat product processing through cavitation. Because of its effectiveness, ultrasonic technology applications have been extended to numerous fields, such as brining, marinating, cooking, and other food processing techniques (7). Research studies have demonstrated that ultrasonic treatment can influence the physicochemical properties of the product, including but not limited to, hardness, water retention, and flavor (8–10). However, the effects of ultrasonic processing parameters, such as time, temperature, power, and other factors, differ depending on the product itself (11). While the ultrasonic technology has been predominantly studied in the ham processing stage, its utilization in the post-ripening phase of ham production remains unexplored (12).

Oral processing, the process of food being masticated, moistened, and enveloped into boluses by saliva and then ingested, plays a crucial role in the formation of food taste (13). Oral food processing involves food dynamics, oral physiology, and sensory psychology and has emerged as a burgeoning subfield of research in food science in recent years (14). Response surface methodology (RSM) is frequently used in food processing to ascertain the most optimal processing conditions (15). This research aims to investigate the physical and chemical properties and sensory attributes of hams as performance indicators, optimize the process parameters of ultrasonic treatment time, temperature, and power using RSM, and subsequently analyze the sensory qualities of the response group following oral processing to select the most optimal process. The results will offer theoretical support for the development of new Mianning hams.

## 2. Materials and methods

### 2.1. Sample

Mianning ham samples were obtained from selected representative factories in Mianning County. The ham was sliced into dimensions of 5 cm × 5 cm × 2 cm, and each sample was supplemented with 1% ultrapure water before vacuum packaging (16). Following vacuum packaging, the ham samples were subjected to ultrasonic treatment using an ultrasonic machine. The treated ham can be used immediately, or refrigerated at 4°C after surface wiping. For subsequent oral processing experiments, the ultrasonically treated ham was boiled in water for 10 min and then cut into 5 ± 1 g cubes (17).

### 2.2. Experimental design

#### 2.2.1. Single-factor test

The primary influential factors of ultrasonic treatment on ham quality are the ultrasonic time, ultrasonic temperature, and ultrasonic power. An analysis was conducted to scrutinize the effects of these three factors (ultrasonic time 40, 60, 80, 100, 120 min, ultrasonic temperature 20, 30, 40, 50, 60°C, and ultrasonic power 100, 125, 150, 175, 200 W) on the desalination rate of Mianning ham using the desalination rate of ham as the index (11).

#### 2.2.2. Response surface testing

Based on the findings from the single-factor test, response surface methodology was utilized to optimize the impact of three factors: ultrasonic time, temperature, and power in Table 1, on the physicochemical and sensory attributes of Mianning ham to enhance its quality. The experimental design employed a three-factor, three-level response surface optimization trial using DesignExpert 8.0.6 software (18). The secondary response surface analysis included linear term effects, interaction term effects, and quadratic term effects, as illustrated in Equation (1).


(1) $$Y=b_{0}+b_{1}\,A+b_{2}\,B+b_{3}\,C+b_{4}\,A\,B+b_{5}\,A\,C+b_{6}\,B\,C+b_{7}\,A^{2}+b_{8}\,B^{2}+b_{9}\,C^{2}\,\left(1\right)$$

**TABLE 1:** Factor levels of response surface tests.

Variables	Level		
	−1	0	1
A Ultrasonic time (min)	60	80	100
B Ultrasonic temperature (°C)	30	40	50
C Ultrasonic power (W)	125	150	175

Y represents the dependent variable, while b_0_ denotes the model constant or intercept. b_1_-b_3_ represent the linear term coefficients, while b_4_-b_6_ depict the interaction term coefficients. Similarly, b_7_-b_9_ represent the quadratic term coefficients. A represents the ultrasonic treatment time, whereas B denotes the ultrasonic treatment temperature, and C represents the ultrasonic treatment power. Lastly, this study aims to optimize the ultrasonic treatment conditions through regression analysis and 3D response surface plots (19).

### 2.3. Physical and chemical testing

#### 2.3.1. Color

The study employed a portable CR400 colorimeter with a light source of D65, an observation angle of 10 degrees, mirror component exclusion mode, and an 8 mm aperture, to determine the brightness L*, redness a*, and yellowness value b* of ham samples, measured from three distinct locations on the sample surface, with each group of samples repeated thrice (20).

#### 2.3.2. pH

The pH value of the ham samples was determined by directly inserting a PH-3C-01 pH meter into the ham samples three times for each group of samples.

#### 2.3.3. Moisture activity

Extracting 3 g of diced sample and spreading it evenly onto the dish designed for measuring water activity, the moisture activity value of the sample was measured using the HD-3A model moisture activity(aw) tester and recorded (21).

#### 2.3.4. Cook loss

The initial weight of the ham was duly accounted for and subsequently subjected to a duration of 10 min in boiling water. Post-cooking, the sample was dried of excess moisture and its weight was re-measured (22).

The calculation of cooking loss rate was then determined through the following formula: percentage of cooking loss = (initial weight−final weight) / initial weight.

#### 2.3.5. Salt content

Begin by weighing precisely 5 g of the sample in a crucible designated for charring. Proceed to establish the ham ash volume at 100 milliliters. Next, transfer 50 milliliters of the test solution to a 250 milliliters conical flask, adding 50 milliliters of water and 1 milliliter of potassium chromate solution (5%). Finally, titrate the mixture with a solution of silver nitrate (0.1%) (23). It is through application of formula (2) that one may accurately calculate the salt content.


(2) $$X=\frac{0.355\times \left(V-0.3\right)}{250}\times 100$$

X is the salt content of the food (%) and V is the volume consumed by the silver nitrate titration.

### 2.4. Texture profile analysis (TPA)

To evaluate the physical properties of the samples, a TAXT Plus mass spectrometer was utilized. The samples were cut into 1 × 1 × 1 cm squares, and the probe was compressed twice, reducing the sample’s original height by 50%, at a speed of 120 mm/min. Subsequently, the hardness (N), adhesion (N*mm), cohesion, elasticity (mm), adhesive viscosity, and chewiness (N) were calculated based on the average of three measurements for each sample (24).

### 2.5. Oral processing

For sensory evaluation, a balanced gender distribution of evaluators consisting of five males and five females, all of whom possess a professional background in food, were formed into a balanced team. It is essential that evaluators oral cavities be free of any abnormalities and they are in good health. They must abstain from smoking, consuming alcohol, or engaging in any other behavior that may affect sensory testing for at least 2 h prior to participating in evaluation. The evaluators within the same group should participate in experiments such as temporal dominance of sensations (TDS), oral treatment analysis, and bolus collection (25).

#### 2.5.1. Bolus collection

The duration of each evaluator’s chewing and subsequent swallowing was recorded as the 100% chewing time point, with the aim of investigating the variations in the physical and chemical properties, as well as the sensory characteristics of ham during oral processing. Boluses from 20, 40, 60, 80, and 100% chewing time points were collected for subsequent experiments. The boluses were either evaluated immediately after collection or preserved by wiping off any surface moisture and refrigerating at 4°C. Masticatory parameters, such as the duration of chewing and the number of chews, were captured via camera during the oral processing stage, and consequently analyzed (26).

#### 2.5.2. Water and saliva content of the bolus

The swallowing points of the bolus were selected at intervals of 20, 40, 60, 80, and 100%. Next, 5 g of the bolus sample is to be weighed and placed in the moisture tester to determine the bolus’ moisture content, which is expressed as a percentage (%).

Saliva content can be calculated through the following formula, as indicated in reference (27): Saliva content = moisture content of the bolus - moisture content of the sample.

#### 2.5.3. Dominance of sensations (TDS)

To that end, it is observed that TDS, a temporal sensory analysis of food products, generates a series of perceptual attributes classified as “dominant” at specific points or times in the dynamic evaluation process. This analysis determines the strength of the dominant rate of sensory attributes over time. Prior to conducting the TDS evaluation, evaluators undergo training in the appropriate sensory aspects of ham, including but not limited to, its hardness, saltiness, juiciness, gumminess, sourness, and tenderness (28). Assess the prevailing sensory characteristics perceived by the evaluators upon commencing mastication of the samples. They have ability to discern identical or contrasting sensory attributes concurrently and desist from sensing any such attributes momentarily prior to swallowing (29). Each assessor supplies three samples, each evaluation session lasting 3 min, and necessitates oral rinsing with pure water prior to every assessment.

### 2.6. Data analysis

The physicochemical, qualitative, and sensory data were subjected to Analysis of Variance (ANOVA) utilizing the SPSS software. Furthermore, T (Tukey’s) test was employed, and the outcomes were deemed highly significant for *p* < 0.01, significant for 0.05 > *p* > 0.01, and insignificant for *p* > 0.05. Each metric was replicated thrice, and the results were plotted using Origin software. Additionally, Design Expert V8.0.6 was utilized for response surface analysis.

## 3. Results and discussion

### 3.1. Single-factor test results

The study presents the impact of ultrasonic treatment’s time, temperature, and power on the desalination rate of Mianning ham, as depicted in Table 2. The desalination rate initially increased and then decreased with an increase in ultrasonic time, ultimately stabilizing at the maximum value after 80 min. Time groups 1, 2, and 3 exhibited significant differences from time groups 4 and 5 (*p* < 0.05), whereas time groups 4 and 5 did not demonstrate any noticeable difference (*p* > 0.05). In conclusion, the single-factor test tentatively selected the ultrasonic time of 80 min. These findings align with Zhang’s study (30). With the rise in ultrasonic temperature, the desalination of Mianning ham exhibited an initial increase followed by a decrease, culminating at its zenith at 40°C. The variations between Temp.1, 2, 3, 4, and 5 were statistically significant (*p* < 0.05). The one-way ultrasonic temperature test was provisionally elected at 40°C. Upon increasing the ultrasonic power, the desalination of Mianning ham displayed a gradual rise, eventually stabilizing at its optimal capacity of 150 W. Notably, there was a significant difference (*p* < 0.05) between power groups 1, 2, and 3 in comparison to power groups 4 and 5. However, there was no notable discrepancy (*p* > 0.05) between power groups 4 and 5. It is worth mentioning that the single-factor tests, for the time being, had selected the ultrasonic power of 150 W.

**TABLE 2:** Effect of different ultrasonic conditions on desalting rate.

Group	Treatment conditions	Desalination rate
Time1	40 min	10.12 ± 0.44^d^
Time 2	60 min	17.00 ± 0.92^c^
Time 3	80 min	24.83 ± 0.72^a^
Time 4	100 min	23.11 ± 0.72^b^
Time 5	120 min	23.40 ± 0.60^b^
Temp. 1	20°C	7.38 ± 0.62^c^
Temp. 2	30°C	12.16 ± 0.75^b^
Temp. 3	40°C	22.23 ± 0.60^a^
Temp. 4	50°C	11.96 ± 0.46^b^
Temp. 5	60°C	3.43 ± 0.86^d^
Power 1	100 W	9.67 ± 0.60^d^
Power 2	125 W	16.85 ± 0.60^c^
Power 3	150 W	24.23 ± 0.46^a^
Power 4	175 W	21.54 ± 1.53^b^
Power 5	200 W	22.23 ± 0.60^b^

Values are expressed as mean ± standard deviation; each 5 rows are grouped together, different rows of lowercase letters in the same group indicate significant differences (*p* < 0.05).

### 3.2. Response surface optimization

Response surface methodology (RSM), is a statistical technique commonly employed in food research to optimize both single and multi-factor response models. The results of the single-factor experiments were carefully analyzed, and the Box-Behnken experimental design was used to conduct the study. The ultrasonic process was optimized to achieve the desired outcome, and the changes in the quality of Mianning ham after ultrasonic treatment were meticulously measured. The experimental protocol and data for the 17 experimental combinations of the Box-Behnken experimental design can be found in Table 3.

**TABLE 3:** Effects of different ultrasonic treatments on physicochemical properties of ham.

Group	Time	Temp	Power	a_*w*_	L*	a*	b*	pH	Desalination rate	Cook loss
	min	°C	W						%	%
0	0	0	0	0.749 ± 0.01^a^	44.17 ± 0.30^a^	11.37 ± 0.30^a^	7.98 ± 0.33^c^	5.73 ± 0.09^a^	/	22.88 ± 1.31^d^
1	60	30	150	0.744 ± 0.01^a^	43.53 ± 0.48^a^	9.51 ± 0.09^b^	9.30 ± 0.46^b^	5.77 ± 0.02^a^	19.55 ± 0.45^c^	26.32 ± 1.14^c^
2	100	30	150	0.732 ± 0.01^b^	43.16 ± 0.25^b^	9.39 ± 0.05^b^	9.23 ± 0.08^b^	5.70 ± 0.08^a^	22.21 ± 0.34^b^	28.79 ± 0.82^b^
3	60	50	150	0.737 ± 0.01^b^	43.26 ± 0.19^b^	9.54 ± 0.28^b^	9.39 ± 0.32^b^	5.78 ± 0.02^a^	20.43 ± 0.34^b^	27.87 ± 0.96^b^
4	100	50	150	0.730 ± 0.01^b^	43.09 ± 0.33^b^	9.51 ± 0.29^b^	9.33 ± 0.14^b^	5.77 ± 0.01^a^	22.21 ± 0.45^b^	28.78 ± 1.46^b^
5	60	40	125	0.727 ± 0.01^c^	43.03 ± 0.45^b^	9.55 ± 0.10^b^	9.27 ± 0.14^b^	5.75 ± 0.09^a^	19.05 ± 0.62^c^	26.86 ± 0.33^c^
6	100	40	125	0.725 ± 0.02^c^	43.63 ± 0.49^a^	9.44 ± 0.23^b^	9.13 ± 0.11^b^	5.78 ± 0.05^a^	21.12 ± 0.45^b^	28.66 ± 2.56^b^
7	60	40	175	0.724 ± 0.01^c^	42.99 ± 0.16^b^	9.31 ± 0.18^b^	9.56 ± 0.14^a^	5.78 ± 0.04^a^	19.84 ± 0.45^c^	26.71 ± 0.43^c^
8	100	40	175	0.717 ± 0.01^d^	43.12 ± 0.34^b^	9.26 ± 0.23^b^	9.49 ± 0.26^b^	5.78 ± 0.04^a^	21.52 ± 0.51^b^	27.98 ± 0.19^b^
9	80	30	125	0.734 ± 0.01^b^	42.93 ± 0.72^b^	9.23 ± 0.19^b^	9.50 ± 0.26^b^	5.78 ± 0.05^a^	20.14 ± 0.45^b^	27.75 ± 0.60^b^
10	80	50	125	0.728 ± 0.01^c^	43.39 ± 0.13^b^	9.33 ± 0.15^b^	9.54 ± 0.27^a^	5.79 ± 0.07^a^	19.35 ± 0.17^c^	26.15 ± 0.54^c^
11	80	30	175	0.730 ± 0.01^b^	42.79 ± 0.60^c^	9.30 ± 0.13^b^	9.49 ± 0.22^b^	5.76 ± 0.09^a^	19.25 ± 0.45^c^	26.69 ± 0.39^c^
12	80	50	175	0.719 ± 0.01^d^	42.91 ± 0.15^b^	9.15 ± 0.33^b^	9.48 ± 0.14^b^	5.75 ± 0.03^a^	20.93 ± 0.51^b^	27.63 ± 0.54^b^
13	80	40	150	0.725 ± 0.01^c^	43.14 ± 0.31^b^	9.55 ± 0.29^b^	9.25 ± 0.19^b^	5.74 ± 0.03^a^	26.16 ± 0.45^a^	30.72 ± 0.51^a^
14	80	40	150	0.727 ± 0.01^c^	43.24 ± 0.29^b^	9.39 ± 0.24^b^	9.26 ± 0.18^b^	5.73 ± 0.01^a^	25.77 ± 0.34^a^	30.86 ± 0.50^a^
15	80	40	150	0.729 ± 0.01^c^	43.47 ± 0.35^b^	9.46 ± 0.10^b^	9.39 ± 0.19^b^	5.75 ± 0.02^a^	26.16 ± 0.45^a^	30.50 ± 0.60^a^
16	80	40	150	0.728 ± 0.01^c^	43.03 ± 0.51^b^	9.49 ± 0.24^b^	9.35 ± 0.27^b^	5.73 ± 0.02^a^	25.96 ± 0.78^a^	30.56 ± 0.66^a^
17	80	40	150	0.729 ± 0.01^c^	43.33 ± 0.01^b^	9.49 ± 0.31^b^	9.22 ± 0.46^b^	5.73 ± 0.03^a^	26.06 ± 0.62^a^	30.59 ± 0.33^a^

Data are expressed as mean ± standard deviation; lowercase letters in different rows in the same column indicate significant differences (*p* < 0.05), as in Tables 4–6. *Expresses the representation of luminance, redness and yellowness values in the colorimetric values.

#### 3.2.1. Water activity, pH

As depicted in Table 3, the water activity (a_*w*_) following ultrasonic treatment was markedly lower in comparison to the control group. The aw of the ham exhibits a declining trend with an escalation in ultrasonic time, temperature, and power. The ham’s muscle fiber structure is damaged by prolonged ultrasonic, prompting the salt-soluble proteins to precipitate onto the ham’s surface, leading to a reduction in its water activity (31).

In the ultrasonic treatment and control groups, there was no substantial alteration in pH (*p* > 0.05) following ultrasound treatment.

#### 3.2.2. Color

Color represents a significant physicochemical parameter in ham that carries a considerable impact on the economic efficiency of this product. Mianning ham L*, a* and b* exhibit marked differences after ultrasonic treatment when compared to the control group (*p* < 0.05). The response group reveals a decrease in L* with increasing ultrasonic power. The *L*-value of the treatment with 175 W power is lower than that in the treatment group with 125 and 150 W. As ultrasonic power increases, a* decreases and b* increases, which can be attributed to the amplification of the cavitation effect that accelerates the oxidation of myoglobin in hams by free radicals generated by water molecules, ultimately leading to a change in color (32). The L*, a*, and b* of hams decrease with an increase in ultrasound time. Prolonged ultrasound destroys the muscle structure of the ham, thus causing myoglobin to undergo destruction, giving the ham a brownish appearance. An increase in ultrasound temperature reduces the ham L* but increases a* and b*. High temperature can lead to the inactivation of some critical enzymes, which in turn reduces the enzymatic reaction and results in a change of color (33).

#### 3.2.3. Desalination and cook loss

The salt content in ham is a crucial determinant influencing consumer preferences. Table 3 displays the desalination rate, which exhibits an initial increase followed by a decrease with increasing ultrasonic treatment time. Notably, the desalination rate of the 80 min treatment group was significantly higher (*p* < 0.05) than that of the 60 min and 100 min treatment groups. Ultrasound expedites the migration of salt from the interior of the ham to its surface. Prolonged ultrasonic treatment, however, results in an elevated concentration of salt on the surface, which in turn impedes salt precipitation. According to reference (34) desalination initially increases and then decreases. Table 3 demonstrates that the increase in ultrasonic temperature leads to the initial increase and subsequent decrease of ham desalination. This occurs because the appropriate temperature accelerates the enzymatic reaction responsible for salt migration from the interior to the surface of the ham. Elevated temperatures, conversely, deactivate particular enzymes and reduce the rate of salt ion migration. As the ultrasonic power increases, the rate of salt ion migration also increases (30).

The steaming loss rate of ham is shown in Table 3, the steaming loss of response group 13, 14, 15, 16 and 17 was significantly higher (*p* < 0.05) than the other treatment groups, Because ultrasonic treatment leads to a significant reduction in the salt ion content of the ham, which affects the solubility properties of the proteins in the ham and increases cooking losses (35).

### 3.3. Texture profile analysis

The Table 4 presents the textural profile analysis of the ham. As the ultrasonic temperature, time, and power increased, the ham hardness decreased significantly (*p* < 0.05). The hardness of ham showed a negative correlation with ultrasonic time. However, prolonged ultrasonic treatment reduced the protease activity and hindered proteolysis, leading to an increase in the hardness of ham. A rise in ultrasound temperature did not entirely trigger the enzymatic activity in the ham at lower temperatures, ultimately leading to an increase in ham hardness. At higher temperatures, the loss of water in the muscle protein of ham resulted in less hardness (36). The increase in ultrasonic power and the amplified cavitation effect of ham caused a loss of internal moisture, leading to a reduction in ham hardness. The elasticity, cohesiveness, and chewiness of the ham were significantly decreased after ultrasonic treatment. The enzymes diffuse more evenly and quickly with the assistance of ultrasound, resulting in enhanced collagen and elastin hydrolysis in the ham muscle fibers. Simultaneously, the gluing effect of ham formed molecular bonds between the precipitated material and muscle, which contributed to an increase in hardness. The decrease in salt concentration limits the solubility of muscle proteins and affects binding capacity and stability (37).

**TABLE 4:** Effect of different ultrasonic treatment on texture of ham.

Group	Time	Temp	Power	Hardness	Springiness	Cohesiveness	Gumminess	Chewiness	Resilience
	min	°C	W	N	mm		N	N*mm	
0	0	0	0	17.95 ± 0.69^a^	0.754 ± 0.03^a^	0.749 ± 0.04^a^	8.55 ± 0.22^a^	4.03 ± 0.49^a^	0.166 ± 0.06^a^
1	60	30	150	8.62 ± 1.51^b^	0.442 ± 0.03^b^	0.481 ± 0.04^b^	4.09 ± 0.49^b^	1.77 ± 0.02^c^	0.145 ± 0.01^b^
2	100	30	150	6.15 ± 0.24^c^	0.346 ± 0.07^c^	0.423 ± 0.03^c^	3.37 ± 0.14^b^	1.48 ± 0.14^c^	0.103 ± 0.02^b^
3	60	50	150	4.99 ± 0.18^d^	0.652 ± 0.04^a^	0.631 ± 0.02^a^	2.78 ± 0.21^c^	3.54 ± 0.65^b^	0.204 ± 0.03^a^
4	100	50	150	3.24 ± 0.21^e^	0.715 ± 0.03^a^	0.581 ± 0.01^b^	3.18 ± 0.58^b^	2.93 ± 0.50^b^	0.186 ± 0.01^a^
5	60	40	125	8.58 ± 0.48^b^	0.438 ± 0.03^b^	0.502 ± 0.02^b^	3.96 ± 0.32^b^	1.53 ± 0.37^c^	0.125 ± 0.02^b^
6	100	40	125	6.65 ± 1.56^c^	0.579 ± 0.07^b^	0.574 ± 0.02^b^	3.41 ± 0.49^b^	1.95 ± 0.06^c^	0.169 ± 0.01^a^
7	60	40	175	8.23 ± 0.28^b^	0.623 ± 0.11^a^	0.598 ± 0.08^a^	4.23 ± 0.46^b^	1.99 ± 0.19^c^	0.181 ± 0.06^a^
8	100	40	175	5.86 ± 0.18^c^	0.664 ± 0.08^a^	0.622 ± 0.02^a^	3.79 ± 0.51^b^	2.76 ± 0.74^b^	0.196 ± 0.02^a^
9	80	30	125	7.20 ± 0.80^c^	0.276 ± 0.04^c^	0.387 ± 0.03^c^	4.14 ± 0.52^b^	2.49 ± 0.59^b^	0.105 ± 0.01^b^
10	80	50	125	3.60 ± 0.23^e^	0.591 ± 0.09^b^	0.596 ± 0.02^a^	3.46 ± 0.43^b^	2.22 ± 0.23^b^	0.195 ± 0.02^a^
11	80	30	175	5.82 ± 0.58^c^	0.608 ± 0.04^b^	0.559 ± 0.05^b^	3.01 ± 0.53^c^	1.77 ± 0.13^c^	0.181 ± 0.01^a^
12	80	50	175	3.48 ± 0.23^e^	0.525 ± 0.01^b^	0.579 ± 0.01^b^	2.91 ± 0.33^c^	1.53 ± 0.16^c^	0.198 ± 0.01^a^
13	80	40	150	4.79 ± 0.45^d^	0.562 ± 0.09^b^	0.542 ± 0.03^b^	2.53 ± 0.32^c^	1.36 ± 0.54^c^	0.173 ± 0.04^a^
14	80	40	150	4.62 ± 0.42^d^	0.563 ± 0.05^b^	0.569 ± 0.04^b^	2.49 ± 0.73^c^	1.36 ± 0.29^c^	0.184 ± 0.02^a^
15	80	40	150	4.79 ± 0.53^d^	0.565 ± 0.03^b^	0.576 ± 0.04^b^	2.66 ± 0.45^c^	1.74 ± 0.24^c^	0.173 ± 0.02^a^
16	80	40	150	4.96 ± 0.39^d^	0.559 ± 0.02^b^	0.581 ± 0.05^a^	2.43 ± 0.44^c^	1.87 ± 0.25^c^	0.230 ± 0.04^a^
17	80	40	150	4.63 ± 0.60^d^	0.563 ± 0.03^b^	0.546 ± 0.04^b^	2.65 ± 0.41^c^	1.68 ± 0.31^c^	0.175 ± 0.04^a^

*Units of chewiness. The superscript letters represent significant differences; the same letters are not significantly different, and different letters represent a limiting difference.

### 3.4. Response surface optimization and modeling

Response surface modeling was executed to investigate the ultrasonic treatment of Mianning ham, and the coefficient of determination (R^2^) along with the lack of fit were selected to examine the adequacy of the response model. The purpose of the lack of fit test is to determine whether the chosen model is in accordance with the observed data. The model is deemed appropriate if the *p*-value of the lack of fit is more than 0.05. The coefficient of determination (R^2^), representing the proportion of variation due to the response surface model, should be more than 95% for a well-fitted model in experimental settings (38). Upon scrutinizing the results, it was observed that the R^2^ values of the response variables, namely desalination rate and hardness, were greater than 95%, and no significant lack of fit was detected. Consequently, the model devised for the desalination rate and hardness of the ham is deemed reasonable.

**FIGURE 1:**
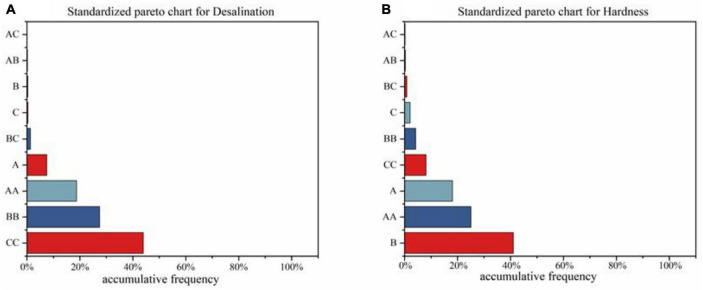
Pareto diagram of the significance of ultrasonic treatment factors on the desalination rate **(A)** hardness **(B)** of Mianning ham.

Table 5 displays the analysis of variance (ANOVA) for the desalination rate and hardness of ham, with A, B, and C denoting the ultrasonic treatment time, temperature, and power, respectively. The Pareto chart (Figure 1) depicts the significance of the effect of each ultrasound treatment factor, with the length of the bars representing their proportion, and the vertical coordinates ordered from top to bottom by degree of influence. The Pareto chart analysis is instrumental in determining the magnitude of the response variable’s impact (39).

**TABLE 5:** Analysis of variance of regression model and significance test of regression coefficients.

Source	Sun of squares	Df	Mean square	F-ratio	*P*-value
**Desalination rate**					
Model	122.72	9	13.64	479.62	<0.0001
A: Time	8.39	1	8.39	295.23	<0.0001
B: Temp	0.39	1	0.39	13.88	0.0074
C: Power	0.44	1	0.44	15.46	0.0057
AB	0.20	1	0.20	6.95	0.0336
AC	0.039	1	0.039	1.37	0.2802
BC	1.52	1	1.52	53.55	0.0002
A^2^	20.86	1	20.86	733.83	<0.0001
B^2^	30.58	1	30.58	1075.70	<0.0001
C^2^	48.98	1	48.98	1722.77	<0.0001
Residual	0.20	7	0.028		
Lack of fit	0.090	3	0.030	1.10	0.4463
Pure error	0.11	4	0.027		
Cor total	122.92	16			
R^2^ = 99.84%					
**Hardness**					
Model	47.34	9	5.26	282.06	<0.0001
A: Time	8.57	1	8.57	459.58	<0.0001
B: Temp	19.47	1	19.47	1044.06	<0.0001
C: Power	0.99	1	0.99	53.31	0.0002
AB	0.13	1	0.13	6.95	0.0336
AC	0.096	1	0.096	5.15	0.0575
BC	0.40	1	0.40	21.28	0.0024
A^2^	11.86	1	11.86	636.16	<0.0001
B^2^	1.98	1	1.98	106.42	<0.0001
C^2^	3.83	1	3.83	205.29	<0.0001
Residual	0.13	7	0.019		
Lack of fit	0.052	3	0.017	0.89	0.5189
Pure error	0.078	4	0.020		
Cor total	47.47	16			
R^2^ = 99.73%					

As depicted in Table 5, the linear impacts of the autonomous variables A, B, and C, along with the interaction effects of AB and BC, as well as the quadratic impacts of AA, BB, and CC, had a considerable (*p* < 0.05) influence on the desalination rate of Mianning ham. Similarly, the linear effects of the independent factors A, B and C, the interaction effects of AB and BC, and the secondary effects of AA, BB and CC significantly (*p* < 0.05) impacted the hardness of Mianning ham. The regression equation for predicting the value of each response variable on changing the response surface variable is as follows.


(3) D⁢e⁢s⁢a⁢l⁢i⁢n⁢a⁢t⁢i⁢o⁢n⁢r⁢a⁢t⁢e=-173.000722+1.01565×A+1.897×B

+1.56356×C-0.00111125×A×B

-0.000197333×A×C+0.00246767

$$\times B\times C-0.00556492\,A^{2}-0.02695\times B^{2}$$

$$-0.00547501\times C^{2}$$

(4) H⁢a⁢r⁢d⁢n⁢e⁢s⁢s=74.171-0.71265×A+0.1322×B-0.49738

×C+0.0009×A×B-0.00031×A×C

$$+0.00126\times B\times C+0.00419625\,A^{2}-0.006865$$

$$\times B^{2}+0.0015256\times C^{2}$$

After generating the model polynomial equations for the dependent and independent variables of interest, the 2 response surface sets are optimally combined. Upon inspection of the Pareto chart (Figure 1A) and the significance analysis in Table 5, it was discovered that ultrasonic time has the most significant impact on the desalination rate, followed by ultrasonic power and finally ultrasonic temperature. Figure 1B and Table 5 reveal the significance analysis of hardness, with ultrasound temperature, ultrasound time, and ultrasound power being the influencing factors in descending order. The R2 values for desalination rate and hardness were 99.84 and 99.73%, respectively. *F*-values were 479.62 and 282.06. The *P*-values were all less than 0.05 and the *P*-values for the lack of fit were greater than 0.05. This indicates that the response surface model is capable of better responding to the predicted values of the regression equation.

**FIGURE 2:**
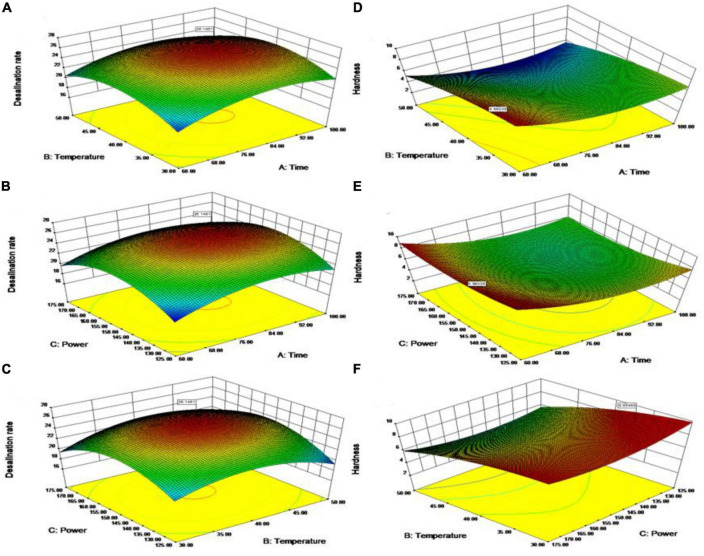
Response surfaces of desalinization rate **(A–C)**, hardness **(D–F)**, after ultrasonic treatment.

A three-dimensional response surface plot of the effect of each factor on the desalination rate and hardness of the ham after ultrasonic treatment was obtained using software analysis (Figure 2).

The graphical representation of the desalination rate surface plot (Figures 2A–C) is displayed. Figure 2A illustrates that as time and temperature increased, while maintaining a constant power of 150 W, the desalination rate also increased, reaching a peak value of 26.15%. Any further increase in time and temperature resulted in a decline in the desalination rate. Furthermore, Table 3 provides evidence that power has a significant impact on the desalination rate of hams. As displayed in Figure 2B, at a fixed temperature of 40°C, the increase in time and power resulted in a decrease in the desalination rate after it reached a maximum of 26.15%. Figure 2C demonstrates that the desalination rate decreases after reaching a maximum of 26.15% when temperature and power are increased at a fixed time of 80 min. The results of the aforementioned experiments demonstrate that time has a crucial linear impact within the studied range of 60–100 min, rather than a quadratic effect. Additionally, the maximum power for the desalination rate at a time of up to 84.56 min was 150.85 W (Figure 2A), and the maximum temperature for the desalination rate at time up to 84.56 min was 40.35°C (Figure 2B).

As shown in Figure 2E, increasing time and power at a fixed temperature of 40°C resulted in a constant decrease in hardness with a maximum value of 8.87 N. Table 4 shows that temperature had a significant effect on the hardness of the ham. Figure 2D shows that at a fixed power of 150 W, the increase in time and temperature causes the hardness to decrease and then increase to a maximum value of 8.87 N. Figure 2F shows that at a fixed time of 80 min, the increase in temperature causes a constant decrease in hardness with a maximum value of 8.87 N. The increase in power leads to a decrease and then an increase in hardness, with a maximum value of 8.87 N. Combined with the analysis of Figures 2D–F and Table 4, the change in temperature in the study range of 30–50°C has an important linear, rather than quadratic, effect of time. When the temperature was 35.37°C, the maximum response of power was 130.08 W (Figure 2D), the maximum response of time was 60.67 min (Figure 2F), and the maximum value of hardness was 8.87 N.

In summary, the optimal desalination rate process conditions after response surface optimization were 84.56 min of ultrasonic time, 40.35°C of ultrasonic temperature, and 150.85 W of ultrasonic frequency, at which the ham desalination rate was 26.15%. The optimal hardness process conditions after response surface optimization were 60.67 min of ultrasonic time, 35.37°C of ultrasonic temperature, and 130.08 W of ultrasonic power, at which time the ham hardness was 8.87 N. In order to select the process conditions with high consumer acceptance, the two corresponding groups mentioned above were subjected to subsequent oral sensory experiments.

### 3.5. Oral processing

#### 3.5.1. Oral processing parameters

The group subjected to oral processing experiments was the ultrasound experimental group, which underwent response surface optimization. The experimental group US1 was treated with an ultrasonic time of 84.56 min, ultrasonic temperature of 40.35°C, and ultrasonic frequency of 150.85 W. On the other hand, the experimental group US2 was subjected to an ultrasonic time of 60.67 min, ultrasonic temperature of 35.37°C, and ultrasonic power of 130.08 W. Both US1 and US2 were cooked in boiling water for 10 min, cut into cubes weighing approximately 5 ± 1 g, and subsequently processed orally.

Mastication is the regular movement of the jaw that grinds food into a bolus that can be reached for swallowing, a process known as oral processing of food. Table 6 describes the masticatory parameters as well as the salivary content during oral processing.

**TABLE 6:** Oral processing parameters of US1 and US2.

Group	Chewing time	Number of chews	Chewing frequency	Chewing rate	Moisture content of bolus	Saliva content
	s		g/s	chew/s	%	%
20%US1	7.94 ± 0.88^e^	8.3 ± 1.25^e^	1.59 ± 0.18^e^	1.04 ± 0.09^b^	58.39 ± 1.06^d^	10.54 ± 1.06^d^
40%US1	15.88 ± 1.75^d^	17.7 ± 1.77^d^	3.18 ± 0.35^d^	1.12 ± 0.09^a^	62.93 ± 1.15^c^	15.08 ± 1.15^c^
60%US1	23.82 ± 2.63^c^	26.1 ± 2.92^c^	4.76 ± 0.53^c^	1.10 ± 0.05^a^	67.31 ± 1.04^b^	19.45 ± 1.04^b^
80%US1	31.76 ± 3.50^b^	35.1 ± 4.20^b^	6.35 ± 0.70^b^	1.11 ± 0.05^a^	69.19 ± 1.17^a^	21.33 ± 1.17^a^
100%US1	39.71 ± 4.38^a^	44.6 ± 5.72^a^	7.94 ± 0.88^a^	1.12 ± 0.04^a^	71.05 ± 1.70^a^	23.19 ± 1.70^a^
20%US2	7.23 ± 0.76^e^	7.0 ± 0.82^e^	1.45 ± 0.15^e^	0.97 ± 0.05^b^	56.16 ± 1.14^d^	10.79 ± 1.14^d^
40%US2	14.45 ± 1.51^d^	16.0 ± 1.41^d^	2.89 ± 0.30^d^	1.11 ± 0.07^a^	60.64 ± 0.95^c^	15.27 ± 0.95^c^
60%US2	21.68 ± 2.27^c^	24.7 ± 2.21^c^	4.34 ± 0.45^c^	1.14 ± 0.05^a^	66.15 ± 1.40^b^	20.78 ± 1.40^b^
80%US2	28.90 ± 3.03^b^	32.3 ± 2.83^b^	5.78 ± 0.61^b^	1.12 ± 0.04^a^	68.38 ± 1.02^a^	23.01 ± 1.02^a^
100%US2	36.13 ± 3.79^a^	40.9 ± 4.12^a^	7.23 ± 0.76^a^	1.13 ± 0.03^a^	70.10 ± 1.38^a^	24.73 ± 1.38^a^

The superscript letters represent significant differences; the same letters are not significantly different, and different letters represent a limiting difference.

Table 6 shows, the greater difference in swallowing time and number of chews between US1 and US2 treatment groups may be related to the salt content of the samples. As shown in Table 3, the desalination rate of US1 was significantly higher (*p* < 0.05) than that of US2. US1 chewed more and chewed longer than US2 and chewed more frequently. This is consistent with Omkar’s study (40). The mastication rates of US1 and US2 were at 1.12 chews/s and 1.13 chews/s, indicating that ultrasound treatment did not result in a change in mastication frequency (41). Prolonged chewing leads to an increased sense of satiety, which helps consumers control food intake for weight loss (42).

During the chewing process, the water content of all bolus increases. The initial moisture content was 47.86 ± 1.01% for US1 and 45.37 ± 1.15% for US2. Even though the initial moisture content of US1 and US2 were different, the difference in moisture content at the 100% chewing time point was not significant. suggesting that saliva compensates to some extent for the difference in initial moisture content during ham chewing, which is consistent with Rizo’s study (43). Saliva content increases with the chewing process. The salivary content of US1 and US2 was similar at 20 and 40% of chewing time points, but US2 had higher salivary content than US1 at 60, 80, and 100% of chewing time points. It is possible that there is little difference in the perception of sensory attributes such as US1 and US2 salinity in the pre-chewing period, resulting in comparable salivary intake in the pre-chewing period. To compensate for the water content of 100% chewing time, so the saliva content of US2 is higher than that of US1 in the late chewing period (40).

#### 3.5.2. Dominance of sensations (TDS)

Temporal dominance of sensations is a sensory description method that requires the evaluator to be able to continuously indicate the dominant sensation. The evaluator’s “dominant sense” was defined as the sense of time to get attention. The TDS is able to collect a range of sensory properties felt at different points in time throughout the chewing process. The sensory characteristics of ham are mainly hardness, saltiness, juiciness, gumminess, sourness and tenderness (44).

**FIGURE 3:**
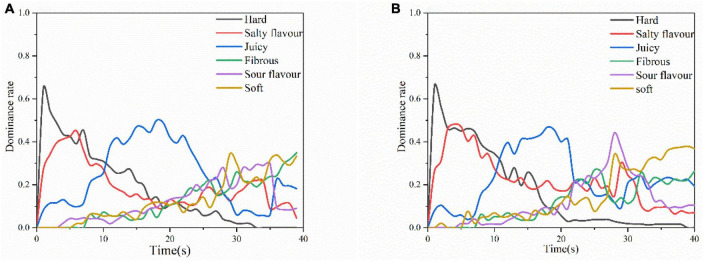
Temporal dominance of sensations (TDS) curves for US1 **(A)** and US2 **(B)**.

Figure 3 depicts the differences in the perception of Mianning ham from first chewing to swallowing US1 and US2 with different ultrasound treatments, as the different ultrasound treatments resulted in differences in ham quality. For US1 (Figure 3A), the sensory dominance within 10 s of chewing was related to hardness; the sensory dominance within 10–25 s of chewing time was juiciness; the sensory dominance within 25–35 s of chewing was softness; and the sensory dominance attribute for the last 5 s of chewing time was gumminess. The salty taste in US1 perception kept decreasing after 5 s of chewing, but the salty taste perception increased slightly again at 25–35 s of chewing time, and the sour taste also peaked at about 35 s of chewing time perception. This may occur because the chewing time is about to reach the swallowing point and the flavor is more easily perceived in the ham (45).

For US2 (Figure 3B), the overall sensory perception was similar to that of US1. However, the dominant rate of hardness is longer than US1 time as shown in Figure 3B. Because the hardness of the US1 ultrasound-treated group was lower than that of US2. US2 salty and sour flavors were perceived higher than US1. US2 sourness dominated the senses for 25–30 s. The overall perceived intensity of saltiness was higher than that of US1 because the desalination rate of US2 was lower than that of US1, which had higher degree of protein hydrolysis, resulting in a saltier and more acidic ham sample. The final swallowing phase softness and gumminess US1 and US2 showed similar perceptions. Because the water content of the food mass was similar at the time of reaching the end point of swallowing, resulting in similar softness and adhesive properties in the TDS images (46).

The response surface optimized US1 and US2 groups were analyzed for oral processing, and the chewing time and chewing frequency were higher in the US1 group than in the US2 group; the US1 group was more likely to produce satiety; and the perception of salty and sour tastes was lower in the US1 group than in the US2 group. In summary, the US1 group was more acceptable than the US2 group, so the US1 group was selected. The ultrasonic treatment conditions were ultrasonic time of 84.56 min; ultrasonic temperature of 40.35°C; and ultrasonic power of 150.85 W.

### 3.6. Validation experiments

On this basis, verification experiments were conducted on the desalination rate and hardness of ham. Using the optimized ultrasonic treatment conditions, the ultrasonic time was 84.56 min; the ultrasonic temperature was 40.35°C; and the ultrasonic power was 150.85 W. The validation results are shown in Table 7, and the predicted values are similar to the validated results. It is shown that the response surface methodology is effective for modeling and optimizing the production operation of ultrasonic treatment of Mianning ham.

**TABLE 7:** Validation experiments.

Response	Predicted	Observed
Desalination rate (%)	26.15 ± 0.00	25.93 ± 0.69
Hardness (*N*)	4.54 ± 0.00	4.48 ± 0.62

## 4. Conclusion

The study found that ultrasound treatment did not affect the pH of the Mianning ham, but it decrease the water activity (a_*w*_) of the ham, particularly with higher ultrasonic time, temperature, and power. Additionally, an increase in ultrasonic power resulted in a decrease in color, as evidenced by a reduction in L* and a*, and an increase in b*. Furthermore, the L*, a*, and b* values of the ham decreased with increasing ultrasonic time. An increase in ultrasound temperature caused a decrease in L* and an increase in both a* and b*. The desalination of the ham initially increased and then decreased with increasing ultrasonic treatment time, temperature, and power. Similarly, the steaming loss of the ham first increased and then decreased with an increase in ultrasonic time, temperature, and power. Finally, the hardness, elasticity, cohesiveness, chewiness and overall texture of the ham gradually decreased as the ultrasonic treatment time, temperature and power increased.

The chewing time and chewing frequency of US1 group were higher than US2, and prolonged chewing was more likely to produce satiety. The TDS curve for US1 was lower than US2 for ham salty and sour perceptions, and US1 was a more suitable choice for consumers. The ultrasonic conditions for optimal acceptance were: ultrasonic time 84.56 min, ultrasonic temperature 40.35°C and ultrasonic power 150.85 W. Verification experiments by this condition yielded a desalination rate of 25.93% ± 0.69% and a hardness of 4.48 N ± 0.62 N. In summary, the model obtained by optimization of ultrasonic desalination technology is suitable for the desalination process of Mianning ham. Thus, providing a theoretical basis for future desalination techniques for dry cured hams.

## Data availability statement

The original contributions presented in this study are included in the article/supplementary material, further inquiries can be directed to the corresponding author.

## Author contributions

JH and LC conceived and wrote the original draft. WW, JZ, YZ, WLW, TB, LJ, and LC reviewed, edited, and revised the manuscript. All authors approved the final version.
